# Outcome Measures in Chronic Rhinosinusitis

**DOI:** 10.1007/s40136-018-0215-3

**Published:** 2018-08-16

**Authors:** Fiona Ting, Claire Hopkins

**Affiliations:** 1 ENT Department, Guy’s Hospital, Great Maze Pond, London, SE1 9RT UK; 2 King’s College London, London, UK

**Keywords:** Outcomes, Outcome measures, PROMs, Chronic rhinosinusitis, Core outcome sets

## Abstract

**Purpose of Review:**

Chronic rhinosinusitis (CRS) has a significant impact on the quality of life of patients and is a high cost burden to both society and patients. There is a variety of objective and subjective outcome measures that exist to assess the effectiveness of interventions. We aim to review current outcome measures available.

**Recent Findings:**

Traditionally, results have focused on objective measures, however, subjective outcome measures are gaining traction as being more important. Outcome measures in chronic rhinosinusitis are currently heterogeneous, thus limiting the impact of meta-analysis of past trial results. The development of a core outcome set may standardize the reporting of outcomes in chronic rhinosinusitis.

**Summary:**

We outline the outcome measures currently available and discuss a proposed core outcome set that may facilitate further value of research on interventions for CRS in adults.

## Introduction

Outcome measures are increasingly important to improving patient-centered care in order to provide better health care delivery. This relies on the promotion and wider usage of assessing outcomes that are important to the patient. Chronic rhinosinusitis (CRS) is a common disorder, can exist with or without polyps, and affects 11% of the UK population [1]. It is a chronic, inflammatory disease that affects patient’s health-related quality of life and productivity. The European Position Paper on Rhinosinusitis and Nasal Polyps [2] defined chronic rhinosinusitis as the inflammation of the nose and paranasal sinuses, characterized by two or more of the following symptoms, persisting for more than 12 weeks:

- Blockage/Congestion: discharge (anterior or post-nasal drip); facial pain or pressure; reduction in smell;
- AND either endoscopic signs of polyps; mucopurulent discharge from the middle meatus; edema or mucosal obstruction primarily in the middle meatus
- AND/OR mucosal changes within the osteomeatal complex and/or sinuses on CT

Treatment options for CRS can be divided into medical and surgery therapy. Medical therapy is the mainstay of treatment but if this fails or complications arise, surgical treatment in the form of endoscopic sinus surgery is considered standard treatment. Patients therefore have several treatment options which may change and evolve throughout their disease progression. The degree of health-related quality of life impairment has been demonstrated to drive patient treatment options. In order to optimize the outcome of interventions, an awareness of different outcome measures and their application is essential.

## Outcome Measures

When considering the impact of treatment options for patients with CRS, it is important to review the types of outcomes that can be measured. Objective measures include endoscopic grading systems, olfactory outcomes, and performance-based measures such as complication rate or recurrence and revision rates for surgery. However, subjective patient-reported outcomes have more recently gained further traction as being a more important factor in outcome measurements in CRS. Subjective measures include patient-reported outcome measures (PROMs) for which there are now a growing number of disease-specific instruments, as well as generic health-related quality of life scores.

The Outcomes Most Important for Patients, Public and Practitioners (OMIPP) project commissioned by Cochrane in 2016 [3] evaluated outcomes from treatments as measured by patients and practitioners for CRS. Only 3% of responses were objective measures with the vast majority of outcomes important to both patients and their treating doctors being subjective. Therefore, primary outcomes in future reviews should focus on subjective outcomes as the main basis for a core outcome set in rhinosinusitis research.

### Subjective Measures

#### Patient-Reported Outcome Measures

Patient-reported outcome measures (PROMs) assess the aspects of care that result in a change in patient health status, productivity, and overall well-being. They are therefore essential to assess whether or not clinicians improve the health of the patient in ways that are meaningful to patients. PROMs may be capable of evaluating the value of care and may predict outcomes such that they may inform the decision as to whether to treat.

A recent systematic review performed by Rudmik et al. [4•] identified 15 PROMs validated for adults with CRS. Of these, the SinoNasal Outcome Test (SNOT-22), the Questionnaire of Olfactory Disorders (QOD), and the Sinusitis Control Test (SCT) contained the highest quality of development and psychometric properties. These three PROMs also evaluated different aspects of CRS including HRQOL and symptoms (SNOT-22), olfaction (QOD), and CRS disease control (SCT).

The SNOT-22 is an outcome measure applicable to both medical and surgical treatments, with a score range of 0–110. It is derived from the SNOT-20 with 2 questions added to measure nasal blockage and sense of taste/smell. Patients rank the severity of 22 symptoms using a 6-point Likert scale. The short form QOD [5] is composed of 25 items, divided into 3 general domains including negative items which assess the degree of suffering, positive items looking at how well patients cope with olfactory dysfunction, and social items which measure response credibility. These items are ranked from 0 to 3 (severe) with a maximum score of 57 points. The SCT assesses the degree of CRS control, looking at how well CRS is controlled using current medical therapies at a specific time. There are 4 questions, each scaled 0 to 4 with an overall total score ranging from 0 to 16. This categorizes patients into well-controlled (1–3), partially controlled (4–11), and uncontrolled (12–16) [6].

However, while there is strong validation and use in the research setting, the main limitations of these PROMs are a lack of items that assess patient preference and value judgements for specific treatment options and there is also a lack of items that assess the impact of common comorbid diseases[4•]. The SNOT-22 has shown the most promise in the clinical setting. Two studies have demonstrated that baseline SNOT-22 scores can be used to predict treatment selection for CRS [7, 8], while a further two studies have shown that baseline SNOT-22 scores can be used to inform patients about their expected HRQOL outcomes after sinus surgery [9, 10].

#### Generic Health-Related Quality of Life Symptom Scores

Generic symptom scores do not focus on a particular disease and assess a range of general physical and emotional symptoms. They allow comparison between different diseases and allow cost-effectiveness assessment in the form of Quality-Adjusted Life Years (QALYs). The EuroQoL five-dimensional questionnaire (EQ-5D) is the only generic instrument that has been validated in the CRS population [4•]. It is a generic measure of a patient’s preference for living in a particular health state and provides health state utility values in the form of QALYs. It measures 5 attributes, each with 3 possible states: mobility, self-care, usual activity, pain/discomfort, and anxiety/depression [11].

#### Productivity and Absenteeism

The effects of CRS on physical and mental health may translate into absenteeism, an absence from work, or presenteeism, which is any deleterious effect on concentration due to CRS whilst at work. Presenteeism is a self-reported measure. The total effect of these can be described as Lost Productive Time. A study by Rudmik [12] assessed the impact of endoscopic sinus surgery on productivity costs which found the total mean productive time due to absenteeism, presenteeism, and loss of household productivity was 75 days per person per year. This equated to an annual productivity cost of $9190 per person.

### Objective Measures

#### Endoscopic Grading Systems

CRS can be assessed by endoscopic evaluation of disease. There are multiple scoring systems; however, the most widely used is that devised by Lund and Kennedy [13]. The Lund-Kennedy endoscopy scoring system grades visual pathologic states within the nose and paranasal sinuses including polyps, discharge, edema, scarring, and crusting. This scoring system is most relevant for CRS with polyposis, for assessment pre- and post-endoscopic sinus surgery. Psaltis et al. [14] describes a modified Lund-Kennedy score which includes polyps, edema, and discharge, and has a high inter-rater and test-retest reliability. Importantly, the modified Lund-Kennedy score correlates well with the SNOT-22. Endoscopic grading systems are very useful in the pre- and post-endoscopic sinus surgery research setting but may be less helpful to monitor non-surgical patients over time.

#### Recurrence/Revision Rate

Revision surgery is an objective, easily measurable outcome; however, defining the exact time of disease recurrence is more difficult and depends on frequency of patient review. The UK National Sinonasal Audit [15] which looked at data collected between 2000 and 2001 remains the benchmark for revision rates due to its long-term follow-up data, and reported approximately 4% of 3128 patients with CRS who had surgery required a revision procedure within 1 year and 11% within 3 years. Of the 52.2% of patients that responded to a 5 year follow-up, 19% of those had had revision surgery. The CRS Epidemiology Study [16] also reports that the highest rates of revision surgery were amongst those patients with CRSwNP and allergic fungal rhinosinusitis. This study, conducted 15 years after the National Sinonasal Audit, found rates of revision remained similar.

#### Complication Rates

The National Sinonasal Audit [15] also reviewed adverse event rates for endoscopic sinus surgery. Major complications include CSF leak and orbital complications including orbital ecchymosis, diplopia or reduction of visual acuity, and significant intra-operative or immediate post-operative hemorrhage. Minor complications include adhesions, infection, minor bleeding, and post-operative pain. Total adverse event rate was 6.6%, mostly relating to minor bleeding. Eleven of 3128 patients had major complications of which seven were orbital complications. Two patients had a CSF leak and a further two had major post-operative hemorrhage requiring return to theaters. There is an increase in complication rates seen with increasing SNOT-22, Lund-Mackay CT scores, and extent of polyposis [15] which indicates that both subjective and objective outcome measures may be able to predict post-operative complication rates.

It must not be forgotten that medical treatment also carries a small, but significant risk of complications. Commonly used topical medications such as intranasal corticosteroids can cause epistaxis, and there is a risk of cardiac arrhythmias with macrolides [17]. Oral corticosteroids are also well known to have multiple adverse effects, ranging from gastritis and disruption to glycemic control, to avascular necrosis and psychosis.

#### Olfactory Tests

Olfactory testing is used more in research studies than in clinical practice and should be able to discriminate between normal function and varying degrees of olfactory dysfunction. One of the most commonly used tests include Sniffin’ Sticks, a test of nasal chemosensory function based on pen-like odor-dispensing devices [18]. Sniffin’ Sticks results are presented as a “TDI score” which is the sum of results obtained for odor threshold, discrimination, and identification measures. Hummel et al. [18] has established normative data based on age groups as odor thresholds decline significantly in relation to age. In addition, women outperform men in all three olfactory tests. This data allows estimation of individual olfactory ability in relation to subject’s age. Absolute hyposmia was defined as the tenth percentile score of 16–35 year old subjects.

#### Core Outcome Sets

Core outcome sets (COS) are an agreed, standardized set of outcomes that can be used to facilitate future meta-analysis of trial results. They are not meant to be restrictive and can include additional outcomes, but are a minimum set of outcomes that should be used in future trials. The use of a COS is supported by the World Health Organization and the Cochrane Group for use in reviews of the effects of healthcare interventions and have been implemented by multiple medical and surgical specialties [19]. The development of core outcome sets has been promoted and supported by the COMET (Core Outcome Measures in Effectiveness Trials) initiative. The use of core outcome sets increases the ability to contribute to systematic reviews, and also aims to reduce outcome reporting bias, where outcomes are measured but left unreported, often in the setting of negative findings.

Hopkins et al. [20••] has developed a COS for chronic rhinosinusitis in adults using COMET guidelines in conjunction with patients, researchers in CRS, and physicians involved in CRS management. The development of the COS involved a systematic review of outcomes used in effectiveness trials to develop a long list of all potential outcomes, followed by sequential rounds of ranking their importance for inclusion in the COS until consensus was achieved, using a Delphi technique. The resulting COS contains 15 items, covering four domains: patient-reported symptoms and QOL, control of disease, impact on daily activity, and acceptability of treatment and side effects (Table 1). Many aspects of the COS are captured by the SNOT-22. However, a modification and re-validation of the SNOT-22 would be required in order to develop a single PROM that could be used in conjunction with endoscopic findings to monitor outcomes over time.

**Table 1:** Final item list for inclusion in Core Outcome Set for CRS, including proposed measurement tools [19]. Permission granted to publish by C Hopkins

Domain	Item	Proposed measurement tool
Patient-reported symptoms and QO	Overall symptom severity	SNOT-22 repeated over time
	Frequency of symptoms	Additional question required to address frequency of symptoms
	Duration of symptoms	
	Duration of treatment effect	
	Sense of smell	
	Runny nose/nasal discharge (anterior or posterior)	
	Nasal obstruction/blockage/congestion	
	Disease-specific quality of life	
Control of disease	Overall control of disease	Need for systemic medication (steroid or antibiotic)
	Need for surgery	
		Progression to surgery
	Endoscopic appearances (including presence/quality of pus, presence and size of polyps, edema, crusting, inflammation)	Lund-Kennedy score
Impact on daily activity	Ability to perform normal activities	SNOT-22 (or specific measures of productivity)
Acceptability of treatment and side effects	Compliance with treatment	Measurement of compliance and side effects
	Acceptability of treatment Side effects of treatment (including medical and surgical)	

## Conclusion

There is currently considerable heterogeneity in the measurement of outcomes in chronic rhinosinusitis which has significant impact on meta-analysis of current and past trial results. The inclusion of a core outcome set in future trials may increase the value of research on interventions for CRS in adults.
